# Supplementary material for: Conditional chemoconnectomics (cCCTomics) as a strategy for efficient and conditional targeting of chemical transmission
Source: eLife. 2024 Apr 30;12:RP91927. doi: 10.7554/eLife.91927 (PMC11060718; doi:10.7554/eLife.91927)
Supplement: Figure 3—source data 3. [file elife-91927-fig3-data3.zip › Figure 3 source data3 part 2.zip!/Figure 3-Source data 3-part2/1062-13-CRISPResso_Report_jt28fhr9.zip!/CRISPResso_on_1062_14/3b.gcaggaaatgagctacctac.Insertion_deletion_substitutions_size_hist.pdf]

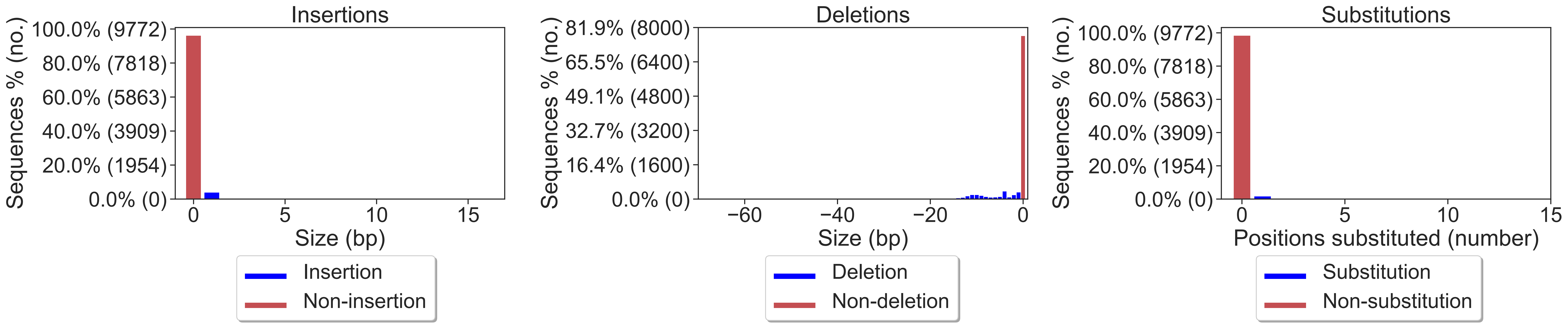
Insertions
Sequences % (no.)
100.0% (9772)
80.0% (7818)
60.0% (5863)
40.0% (3909)
20.0% (1954)
0.0% (0)
0
5
10
15
Size (bp)
Insertion
Non-insertion
Deletions
Sequences % (no.)
81.9% (8000)
65.5% (6400)
49.1% (4800)
32.7% (3200)
16.4% (1600)
0.0% (0)
−60
−40
−20
0
Size (bp)
Deletion
Non-deletion
Substitutions
Sequences % (no.)
100.0% (9772)
80.0% (7818)
60.0% (5863)
40.0% (3909)
20.0% (1954)
0.0% (0)
0
5
10
15
Positions substituted (number)
Substitution
Non-substitution
